# Automatic berthing of unmanned surface vessels with predetermined performance

**DOI:** 10.1038/s41598-024-56188-x

**Published:** 2024-03-19

**Authors:** Qiwen Wang, Qiang Zhang, Enrui Zhao, Yang Liu, Yan Zhang

**Affiliations:** School of Navigation of Shipping, Shandong Jiaotong University, Weihai, 264200 China

**Keywords:** Engineering, Mathematics and computing

## Abstract

To solve the problem of ship automatic berthing control due to unknown time-varying disturbance and dynamic uncertainty of model parameters, an automatic berthing control law based on predefined performance time function is proposed. First, a predefined performance time function is designed and coupled with tracking error to achieve the predetermined performance of tracking error. Secondly, radial basis function neural network is used to approach the dynamic uncertainty of ship model parameters, and the complex uncertainty of model parameters and unknown time-varying disturbance is represented by linearized parameter form with single virtual parameter, which makes the calculation simple and easy to implement in engineering. On this basis, the reverse step control law is designed. Thirdly, the stability of the system is proved based on Lyapunov stability theory. Finally, the simulation results show that the control law can make the ship reach the desired position and heading angle, and realize the automatic berthing of the ship. The control law and berthing controller designed in this paper have good applicability and robustness, which provides a theoretical basis for the subsequent control research of surface intelligent ships.

## Introduction

Automatic vessel berthing is an important research direction of unmanned vessel control. In addition to common wind, wave and current interference, vessels are also affected by shore wall effect and shallow water effect^1^ during automatic berthing, which makes automatic berthing control more difficult than common tracking control. Domestic scholars have adopted a variety of methods, including backstep method^2^ and neural network^3^, to study automatic berthing of vessels, and achieved good results.

Ahmed et al.^4^ used reconstructed mathematical data to train neural networks and proved that the neural network control law can realize automatic berthing control of vessels under gusty wind conditions. Zhang et al.^5^ designed a convenient nonlinear neural network controller for vessels. When processing large sample data, they selectively adjusted the extraction frequency of effective information data, optimized the control effect, shortened the training time, and reduced the calculation dimension and calculation load while maintaining the control effect. Yupeng et al.^6^ used the relative position in the berth coordinate system to train the neural network controller, which expanded the scope of application of the neural network controller and reduced the cost of neural network training. Nguyen et al.^7^ used adaptive interaction technology to train neural networks online without any teaching data and offline training stage. Wang et al.^8^ introduced dynamic surface technology into the inverse design of control systems for nonlinear systems, simplifying the controller design process and eliminating the problem of “differential explosion” in the design. However, currently, the application of neural network algorithms for automatic berthing of ships mostly remains in the simulation testing stage. However, in practical applications, the real-time performance of this method is difficult to ensure, and it is difficult to obtain samples for parameter training. However, the allowed control convergence time for ships during berthing is relatively short, making it difficult for artificial neural network control methods to be applied to the actual berthing process of intelligent ships.

At present, most studies on surface vessels focus on the steady-state performance, and few studies on the transient performance. In this paper, due to the limited berth space when a vessel enters a port, predetermined performance control with limited tracking error is needed. The tracking position is required to be within a certain range, which has certain requirements for the transient performance of the controller. The control performance of the above control methods is limited to converging to a small residual set, and the size of the residual set depends on the design parameters and some unknown bounded terms, which cannot provide guaranteed transient performance in the instant of time. And in practical engineering, it is often required that the proposed control scheme meet certain quality performance indicators such as overshoot, convergence speed, and steady-state error. In the presence of unknown uncertainties and external disturbances in the system, the issue of predetermined performance is also extremely challenging and difficult to achieve. To solve such problems, Hua et al.^9^ designed a non-singular fast-integrating terminal sliding mode controller based on E-BLF based on an exponential obstacle Lyapunov function, which made the trajectory tracking error of underwater vehicles converge to zero in a finite time and meet the predetermined performance requirements. Shao et al.^10^ proposed an adaptive pre-defined performance neural control scheme. The uncertainty and unknown dynamics of the model are estimated by using neural network. An improved predetermined performance function is designed to ensure the transient behavior of tracking errors. Yang et al.^11^ added a Nussbaum-type function to the predefined performance controller to estimate the unknown control direction, ensuring the bound ability of all signals in the closed-loop system. Zhang et al.^12^ used fuzzy logic systems to identify unknown nonlinear systems and established a fuzzy state observer to estimate the uncertain state of the system. A new adaptive fuzzy output feedback control method is proposed under the framework of backstepping control design and the pre-defined performance technology. Lu et al.^13^ solved the non-affine structure in the state by introducing a new coordinate transformation; The designed Lyapunov function makes the system meet the predetermined performance requirements. Ma et al.^14^ designed a vessel trajectory tracking controller with preset performance based on dynamic surface technology. The introduction of dynamic surface technology solves the problem of computational expansion in the traditional backstep method. Based on the traditional preset performance control, Wu^15^ proposed a new performance function with explicit terminal convergence time, and converted the bench-based AUV control system into a new error system by using the new performance function and error transformation. Then, a perturbation observer is designed to estimate the lumped uncertainties caused by current disturbance, model uncertainty and propeller failure, and a whole control algorithm is constructed by using the estimated results. Jiao et al.^16^ introduced a performance function with constraints to design the controller. Firstly, vessel trajectory tracking errors with inequality constraints are converted into equivalent unconstrained errors, and then the controller is designed by combining the converted errors with sliding mode control to ensure the rapidity and high precision of vessel trajectory tracking control. Table 1 shows the research methods and problems currently adopted by different researchers.

**Table 1:** Current research status of automatic berthing control for ships.

Researcher	Research method	Problem	Experimental methods
Ahmed and Hasegawa^4^, Zhang et al. ^5^, Jia et al.^6^, Nguyen and Jung	Neural networks	Automatic berthing control of ships under wind and wave interference	Simulation
^8^	Neural networks	eliminating the problem of "differential explosion" in the design	Simulation
Hua et al.^9^	Barrier Lyapunov function	Stable control within a finite time	Simulation
Zhang et al.^12^	Fuzzy state observer, Predefined performance techniques	The uncertain state of the system	Simulation

At present, there are still many problems that need to be solved in the study of automatic berthing control of ships, such as: the accuracy of the ship model used is low, the ship berthing path cannot be determined, the stabilization time required by the system is long, and it does not meet the requirements of practical applications.

**Figure 1:**
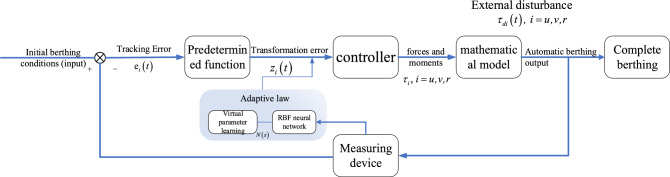
Automatic berthing control process.

To solve the above problems, based on the existing research, this paper proposes an automatic berthing control method based on predetermined performance time function for ship automatic berthing research. Firstly, a predefined performance time function is designed, and then the tracking error is converted into the conversion error by error transformation. On this basis, a reverse step controller is designed. Then neural network is used to overcome the uncertainty of model parameters. The adaptive law is introduced to estimate the boundary of the sum of the external unknown interference and the model approximation error. In order to verify the effectiveness of the method, simulation tests are carried out on the control scheme designed in this paper using simulation software. It can be verified that the control scheme provided in this paper can keep the tracking error stable within the designed range and optimize the transient performance during berthing. Figure 1 shows the automatic berthing control process under predetermined performance.

The main innovation of this article lies in:

1. In this paper, a new nonlinear transformation method is designed, which couples the tracking error and the newly constructed predefined performance time function into a new nonlinear term, which ensures the stability time and tracking accuracy of the control scheme
2. This paper introduces the pre-defined performance control method into the asymmetric automatic berthing process and expands the application scenarios of the pre-defined performance control method.

The remainder of this article is organized as follows. In the second part, the three degrees of freedom surface ship model and the pre-preparation of the predetermined performance function are described. In the third part, a predetermined performance time function is proposed and the berthing error is converted into the conversion error. In the fourth part, an automatic berthing control scheme for ships with predetermined performance is proposed. In the fifth part, simulation results are given to prove the effectiveness of the control scheme. The sixth part lists the conclusions and the direction of future research.

## Problem formulation

Automatic the kinematic and kinetic model of three degrees of freedom surface vessels^17^ is

1 $$\left\{\begin{array}{l}\dot{\eta }=J(\psi )\upsilon \\ M\dot{\upsilon }+C(\upsilon )\upsilon +D\upsilon +\Delta f=\tau +{\tau }_{d}\end{array}\right.$$

In the formula, $$\eta ={\left[\begin{array}{ccc}x& y& \psi \end{array}\right]}^{T}$$ is the actual position of the vessel in the geodetic coordinate system $\left(x,y\right)$ and a vector consisting of the bow angle $\psi$;$$\upsilon ={\left[\begin{array}{ccc}u& v& r\end{array}\right]}^{T}$$ is the velocity of the vessel in the attachment frame, Among them, it is the forward speed $u$,the horizontal drift speed $v$,$r$ is the yaw angular velocity; $J(\psi )$ is the coordinate transformation matrix, Its expression is $$J(\psi )=\left[\begin{array}{ccc}{\text{cos}}\psi & -{\text{sin}}\psi & 0\\ {\text{sin}}\psi & {\text{cos}}\psi & 0\\ 0& 0& 1\end{array}\right]$$; $$\tau ={\left[\begin{array}{ccc}{\tau }_{u}& {\tau }_{v}& {\tau }_{r}\end{array}\right]}^{T}$$ is the control vector composed of forward force ${\tau }_{u}$, lateral drift force ${\tau }_{v}$, and yaw moment ${\tau }_{r}$ for the vessel's control input; $${\tau }_{d}={\left[\begin{array}{ccc}{\tau }_{du}& {\tau }_{dv}& {\tau }_{dr}\end{array}\right]}^{T}$$ is the lateral interference force caused by wind, wave and flow under the attachment coordinate system ${\tau }_{du}$, longitudinal interference force ${\tau }_{dv}$ and the bow interference moment ${\tau }_{dr}$ composition of the disturbance vector of the unknown external environmen $$M=\left[\begin{array}{ccc}{m}_{11}& 0& 0\\ 0& {m}_{22}& 0\\ 0& 0& {m}_{33}\end{array}\right]$$ is the inertial parameter matrix of the additional mass; $$C(\upsilon )=\left[\begin{array}{ccc}0& 0& -{m}_{22}v\\ 0& 0& {m}_{11}u\\ {m}_{22}v& -{m}_{11}u& 0\end{array}\right]$$ is the Coriolis and centripetal force matrices; $$D=\left[\begin{array}{ccc}{d}_{11}& 0& 0\\ 0& {d}_{22}& 0\\ 0& 0& {d}_{33}\end{array}\right]$$ is the damping parameter matrix; $$\Delta f={\left[\begin{array}{ccc}\Delta {f}_{u}& \Delta {f}_{v}& \Delta {f}_{r}\end{array}\right]}^{T}$$ is the uncertain part of the vessel model. In the intelligent vessel mathematical model described in (1), $J(\psi )$ and $M$ has the following properties.

### Property 1

$J(\psi )$ is orthogonal,approach $\Vert J(\psi )\Vert =-1$, ${J}^{-1}(\psi )={J}^{T}(\psi )$;

### Property 2

$$\dot{J}(\psi )=rJ(\psi )E$$, $${\dot{J}}^{T}(\psi )=-rE{J}^{T}(\psi )$$, where $$E=\left[\begin{array}{ccc}0& -1& 0\\ 1& 0& 0\\ 0& 0& 0\end{array}\right]$$;

### Property 3

$M$ is a positive definite symmetric matrix and satisfy $\underline{m}{I}_{3}\le M\le \bar{m}{I}_{3}$, among $\underline{m}$ and $\bar{m}$ are the normal number, ${I}_{3}$ is a three-dimensional identity matrix. As is known from properties 1 and 2 $\dot{J}(\psi ){\dot{J}}^{T}(\psi )=-{r}^{2}{E}^{T}E=0$. In addition, due to $M$ is a constant matrix,so $\underline{m}$ and $\bar{m}$ respectively The minimum and maximum eigenvalues of $M$.

### Assuming 1

${\tau }_{d}$ is unknown but bounded, meaning that there is an unknown normal number ${d}_{i}(i=1,2,3)$ that makes ${\tau }_{d,i}\le {d}_{i}$.

### Assuming 2

Matrix $M,C(\upsilon ),D(v)$ are all unknown matrices.

### Assuming 3

The velocity $v$ is unknown.

In practice, because the energy of the environmental disturbance is limited, the environmental disturbance acting on the vessels body is not easily recognized and limited. The matrix $M,C(\upsilon ),D(v)$ contains mass, additional mass and inertia, and hydrodynamic parameters related to environmental conditions and vehicle mobility and the vehicle itself. Therefore, it is difficult to obtain accurate values for these parameters. In general, the speed of a vessel is usually obtained by the sensors carried by the smart vessel, and the measured value cannot be input to the feedback control loop due to the sensor failure or measurement noise. Thus, Assuming 1–3 is reasonable.

The control goal of this paper is to design a robust adaptive neural network output feedback control for water surface intelligent vessels under hypothesis 1–3. Making it possible to ensure that the berthing error $\eta -{\eta }_{d}$ has predefined transient and steady-state properties, and that $\eta -{\eta }_{d}$ converges to the predefined residue set in a user-defined time frame.

## Previous preparation

### Definition 1

For any vector $\delta \in {R}^{n}$, the matrix $Tanh(\delta ):{R}^{n}\to {R}^{m\times n}$ can be defined as

2 $$Tanh(\delta )=diag(\mathit{tanh}({\delta }_{1})\ldots \mathit{tanh}({\delta }_{n}))$$

where, $diag(\dot)$ Represents a diagonal matrix.

### Lemma 1

^18^For any $\upsilon >0$ and any $\upsilon \in R$, then the following inequality holds.

3 $$0\le \left|\upsilon \right|-\upsilon \mathit{tanh}(\frac{\upsilon }{\omega })\le 0.2785\omega $$

### Lemma 2

^19^: For any $a,b \in R^{2}$, the following inequality holds.

4 $$ab\le \frac{{\iota }^{p}}{p}{\left|a\right|}^{p}+\frac{1}{{\iota }^{p}q}{\left|b\right|}^{q}$$

where $\iota >0$, $p>1$, $q>1$ are constants that satisfy $(q-1)(p-1)=1$.

### Lemma 3

For the continuous function ${\Omega }_{X}\in {R}^{n}$ defined on the compact set $f(X):{R}^{n}\to R$, there exists an RBF neural network function satisfied

5 $$f(X)={W}^{T}\xi (X)+\varepsilon $$

where $W={\left[{W}_{1},\ldots,{W}_{n}\right]}^{T}$ is the ideal weight vector, $\xi (X)={\left[{\xi }_{1}(Z),\ldots,{\xi }_{n}(Z)\right]}^{T}$ is the basis function vector for $\Vert \xi (X)\Vert \le \sqrt{n},n>1$ , $\varepsilon$ is the approximation error. The base function ${\xi }_{i}(X),i=1,\ldots,n$ is a Gaussian function, ${\xi }_{i}(X)=\mathit{exp}\left[-{\left(X-{\iota }_{i}\right)}^{T}\left(X-{\iota }_{i}\right)/{\omega }_{i}^{2}\right]$, Where ${\iota }_{i}={\left[{\iota }_{i,1},\ldots,{\iota }_{i,n}\right]}^{T}$ is the width of the accepted field, and ${\omega }_{i}$ is the width of the Gaussian function. There are also position constants ${W}_{m}$ and $\bar{\varepsilon }$, such that $\Vert W\Vert \le {W}_{m}$ and $\Vert \varepsilon \Vert \le \bar{\varepsilon }$.

### Lemma 4

Consider the RBF neural network with a Gaussian basis function, if $\widehat{X}$ is the input vector of the neural network, $\widehat{X}-X=\delta k$, where $\delta$ is the normal number, $k$ is the bounded vector, there is a bounded function vector ${k}_{x}$, making the

6 $$\zeta (\widehat{X})-\zeta (X)=\delta {k}_{x}$$

where, ${k}_{x}$ satisfies $\Vert {k}_{x}\Vert \le \bar{k}$ and $\bar{k}$ is the normal number.

## Performance function and tracking error transformation

According to formula (1), the dynamic equation of smart ship can be rewritten as

7 $$\dot{\eta }=J(\psi )\upsilon , \dot{\upsilon }=F(v,\tau )+{\tau }_{d}^{*}$$

where $F(v,\tau )=-{M}^{-1}(C(\upsilon )\upsilon +D\upsilon -\tau )$.According to Property 2, is unknown and can be approximated by the RBF neural network in Lemma 3

8 $$F(X)={W}_{0}^{T}\xi (X)+{\varepsilon }_{0}$$

where $X={\left[{v}^{T},{\tau }^{T}\right]}^{T}$, ${W}_{0}=diag({W}_{\mathrm{0,1}}^{T},{W}_{\mathrm{0,2}}^{T},{W}_{\mathrm{0,3}}^{T})$ , ${W}_{0,i}=\left[{W}_{0,i1},\ldots,{W}_{0,in}\right]$ is an ideal weight matrix. $\xi (X)={\left[{\xi }_{1}(X{)}^{T},{\xi }_{2}(X{)}^{T},{\xi }_{3}(X{)}^{T}\right]}^{T}$, ${\xi }_{i}(X)={\left[{\xi }_{i1}(X),\ldots,{\xi }_{in}(X)\right]}^{T}$ is the basis vector. The approximate error vector ${\varepsilon }_{0}$ has $\Vert {\varepsilon }_{0}\Vert \le {\bar{\varepsilon }}_{0}$, where ${\varepsilon }_{0}$ is a constant greater than 0.

Performance function and berthing error conversion

### Definition 2

The smooth function $\sigma (t):{R}_{+}\to {R}_{+}$ is a performance function and satisfies the following conditions:

1. $\sigma \left(t\right)$is positive definite and decreasing in one direction.
2. ${\mathit{lim}}_{t\to \infty }\sigma (t)={\sigma }_{\infty }>0$, where ${\sigma }_{\infty }$ is a scale.

Inspired by Definition 2, we introduce the following new concept, called predefined time performance function (PTPF)

### Definition 3

A smooth function (t) that satisfies the following condition: $\sigma (t):{R}_{+}\to {R}_{+}$ can be said to be PTPF.

1. $$\sigma (t)>0,\forall t\ge 0$$
2. $$\dot{\sigma }\left(t\right)\le 0,\forall t\ge 0$$
3. ${\mathit{lim}}_{t\to {T}_{f}}\sigma (t)={\sigma }_{{T}_{f}}>0$, where the scale ${\sigma }_{{T}_{f}}$ and the time constant ${T}_{f}$ can be customized.
4. $$\sigma \left(t\right)={\sigma }_{{T}_{f}},\forall t\ge {T}_{f}$$

According to Definition 3, PTPF $\sigma (t)$ in this paper is constructed^20^ as follows:

9 $$\sigma (t)=\frac{1}{ht+l}\psi (t)+{\sigma }_{{T}_{f}}$$

10 $$\psi (t)=\left\{\begin{array}{ll}\frac{1}{2}\mathit{cos}\left(\frac{\pi t}{{T}_{f}}\right)+\frac{1}{2},&\quad t<{T}_{f}\\ 0, &\quad  t\ge {T}_{f}\end{array}\right.$$

where, $h$ and $l$ are design parameters, and $\sigma (t)$ has the following properties:

1. $$\sigma \left(0\right)={l}^{-1}+{\sigma }_{{T}_{f}}>{\sigma }_{{T}_{f}}$$
2. $${\mathit{lim}}_{l\to 0}\sigma \left(0\right)=+\infty $$

According to the PPC method^21^, if the berthing error ${e}_{1}=\eta -{\eta }_{d}$ meets $\forall t>0$, the predefined performance can be achieved:

11 $$-{\sigma }_{i}<{e}_{1,i}<{\sigma }_{i},\quad i=1,2,3$$

where ${e}_{1,i}$ is the $i$ th element of ${e}_{1}$, and ${\sigma }_{i}=\sigma (t)$ According to the inequality of $\sigma (t)$, the ${e}_{1,i}(0)$ inequality of $\left|{e}_{1,i}(0)\right|<{\sigma }_{i}(0)$ is always true for suitably small $l$ for bounded.

The predetermined performance function plays an important role in the upper bound of the automatic berthing error ${e}_{1,i}$. In this paper, four design parameters are used to describe the control performance of intelligent ships. The parameters ${h}_{i}$ and ${l}_{i}$ represent the decay rate of ${\sigma }_{i}$ and the initial value ${\sigma }_{i}(0)$, respectively. Where ${l}_{i}$ must meet $\left|{e}_{1,i}(0)\right|<{\sigma }_{i}(0)$.The parameter ${\sigma }_{{T}_{f,i}}$ represents the predefined convergence set of ${e}_{1,i}$ at steady state ${T}_{f,i}$ stands for predefined error enters and stays at the stable time in the interval $(-{\sigma }_{{T}_{f,i}},{\sigma }_{{T}_{f,i}})$. It should be noted that ${T}_{f,i}$ should be set according to the actual requirements and operability of smart ships. In actual operation, the maneuverability of intelligent ship is mainly determined by maneuverability indexes such as pushing moment, turning moment, turning initial diameter and turning final diameter. These indexes can be obtained by maneuvering tests such as steering maneuvers, zigzag maneuvers and free maneuvers.

In other literature, the designed automatic berthing control law for intelligent ships requires an exact initial condition ${e}_{1}(0)$ to determine the performance function $\sigma (t)$.

In practice, however, it is difficult to obtain accurate initial condition information about ${e}_{1}$, and sometimes only cursory information about ${e}_{1}(0)$ is available. In this paper, using the above relation and the newly constructed function (11), we can choose the design constant l to relax the requirement for the exact initial condition ${e}_{1}(0)$.

To ensure that the automatic berthing tracking error ${e}_{1}$ satisfies the inequality of $\forall t\ge 0$, we introduce the following nonlinear transformation:

12 $${z}_{i}=\frac{{\sigma }_{i}^{2}{e}_{1,i}}{{\sigma }_{i}^{2}-{e}_{1,i}^{2}}$$

where ${z}_{i}$ is the transformation variable. From the above formula, we can get ${z}_{i}={\mathit{lim}}_{{e}_{i}\to +{\sigma }_{i}}=+\infty$, ${z}_{i}={\mathit{lim}}_{{e}_{i}\to -{\sigma }_{i}}=-\infty$. Therefore, if ${z}_{i}\in {L}_{\infty }$ and $\left|{e}_{1,i}\right|<{\sigma }_{i}(0)$ can be guaranteed, the error can be assumed to be stable within our predefined range.

## Control law design and stability analysis

In this section, for the theoretical surface intelligent ship with dynamic uncertainty and external interference, based on the designed berthing error transform (12), adaptive neural network technology and vector backstepping design tool are used to design an adaptive neural output feedback automatic berthing control law. The whole design process consists of two steps.

Step 1: Find the derivative of ${z}_{i}$ with respect to time yield

13 $${\dot{z}}_{i}={\psi }_{i}({\dot{e}}_{1,i}-{\phi }_{i}{e}_{1,i})$$

where, ${\psi }_{i}=\frac{{\sigma }_{i}^{2}({\sigma }_{i}^{2}+{e}_{1,i}^{2})}{{\left({\sigma }_{i}^{2}-{e}_{1,i}^{2}\right)}^{2}}$, ${\phi }_{i}=\frac{2{\dot{\sigma }}_{i}{e}_{1,i}^{2}({\sigma }_{i}^{2}-{e}_{1,i}^{2}{)}^{2}}{{\sigma }_{i}({\sigma }_{i}^{2}+{e}_{1,i}^{2})}$.Taking into account the properties of ${\sigma }_{i}$ above, it can be determined that as long as Eq. (11) holds, ${\psi }_{i}>0$.

Further, let ${e}_{1}$ take the derivative of time, using Eqs. (1) and (13), we can get

14 $$\dot{z}=\psi (J(\varphi )v-{\dot{\eta }}_{d}-\phi {e}_{1})$$

where, $$z={\left[{z}_{1},{z}_{2},{z}_{3}\right]}^{T}$$, $$\psi =diag({\psi }_{1},{\psi }_{2},{\psi }_{3})$$, $$\phi =diag({\phi }_{1},{\phi }_{2},{\phi }_{3})$$.

The design virtual control law $\alpha$ is as follows:

15 $$\alpha ={J}^{T}(\varphi )(-{\psi }^{-1}{\beta }_{1}z+\phi {e}_{1}+{\dot{\eta }}_{d})$$

where, ${\beta }_{1}={\beta }_{1}^{T}\in {R}^{3\times 3}$ is the positive definite design matrix.

Step 2: The error vector ${e}_{2}\in {R}^{3}$ is defined as follows:

16 $${e}_{2}=\upsilon -\alpha $$

The derivative of ${e}_{2}$ is obtained by using Eqs. (4) and (16)

17 $$M{\dot{e}}_{2}=M\dot{v}-M\dot{\alpha }=-C(v)v-D(v)v-M\dot{\alpha }+\tau +{\tau }_{d}$$

According to Eqs. (12) and (13) and Property 2, $\dot{\alpha }$ can be further rewritten as

18 $$\alpha =-rE\alpha +{J}^{T}(\varphi )\left\{-{\dot{\psi }}^{-1}{\beta }_{1}z-({\beta }_{1}-\phi )\left(J(\varphi )\nu -{\dot{\eta }}_{d}\right)+({\beta }_{1}\phi +\dot{\phi }){e}_{1}+{\ddot{\eta }}_{d}\right\}$$

Let $H(Z)=-C(v)v-D(v)v-M\dot{\alpha }$, where $Z={\left[{\nu }^{T},{\alpha }^{T},{e}_{1}^{T},{\sigma }_{i}^{T},{\dot{\sigma }}_{i}^{T},{\ddot{\sigma }}_{i}^{T}\right]}^{T},i=1,2,3.$ But $H(Z)$ cannot be used directly in controller design. Since $C(v),D(v),M$ are all unknown according to hypothesis 2, in order to solve this problem, RBF neural networks can be used to approximate the unknown function vector $H(Z)$, which can be obtained

19 $$H(Z)={W}_{c}^{T}\xi (z)+{\varepsilon }_{c}$$

where, ${W}_{c}=\left({W}_{c,1}^{T},{W}_{c,2}^{T},{W}_{c,3}^{T}\right)$, $\xi (z)={\left[{\xi }_{1}(z{)}^{T},{\xi }_{1}(z{)}^{T},{\xi }_{1}(z{)}^{T}\right]}^{T}$. ${\varepsilon }_{c}$ is the approximate error vector of the neural network, and ${\varepsilon }_{c}\in {R}^{3}$ satisfies $\Vert {\varepsilon }_{c}\Vert \le {\bar{\varepsilon }}_{c}$, where ${\bar{\varepsilon }}_{c}$ is a constant.

Let ${d}_{c}={\varepsilon }_{c}+{\tau }_{d}$, and then let assuming 1 and lemma 3 have an unknown vector.

$\theta ={\left[{\theta }_{1},{\theta }_{2},{\theta }_{3}\right]}^{T}$ leads to ${\theta }_{i}>\left|{d}_{c,i}\right|,(i=1,2,3)$,${\theta }_{i}$ is a normal number. According to lemma 1, we can get:

20 $${e}_{2}^{T}\left[{d}_{c}-Tanh(\frac{{e}_{2}}{\sigma })\theta \right]\le {\theta }^{T}\left[\langle {e}_{2}\rangle -Tanh\left(\frac{{e}_{2}}{\sigma }\right){e}_{2}\right]\le 0.2785{\sigma }^{T}\theta $$

where, $$\langle {e}_{2}\rangle ={\left[\left|{e}_{\mathrm{2,1}}\right|,\left|{e}_{\mathrm{2,2}}\right|,\left|{e}_{\mathrm{2,3}}\right|\right]}^{T}$$.

On this basis, the automatic berthing control law of surface intelligent ship is designed

21 $$\tau =-{\beta }_{2}{\widehat{e}}_{2}-{J}^{T}(\varphi )\psi z-{\widehat{W}}_{c}^{T}\xi (\widehat{Z})-Tanh(\frac{{\widehat{e}}_{2}}{\sigma })\widehat{\theta }$$

Adaptive law

22 $${\dot{\widehat{W}}}_{c}={\Lambda }_{c}\left(tr(\xi (\widehat{Z}){\widehat{e}}_{2}^{T})-\kappa {\widehat{W}}_{c}\right)$$

23 $$\dot{\widehat{\theta }}=\Gamma \left[Tanh\left(\frac{{\widehat{e}}_{2}}{\sigma }\right){\widehat{e}}_{2}-\chi \widehat{\theta }\right]$$

where,$$\widehat{ Z}={\left[{\nu }^{T},{\alpha }^{T},{e}_{1}^{T},{\sigma }_{i}^{T},{\dot{\sigma }}_{i}^{T},{\ddot{\sigma }}_{i}^{T}\right]}^{T}$$, $${e}_{2}=v-\alpha, {\beta }_{2}={\beta }_{2}^{T}\in {R}^{3\times 3},{\Lambda }_{c}={\Lambda }_{c}^{T}\in {R}^{3\times 3}$$ and

$\Gamma ={\Gamma }^{T}\in {R}^{3\times 3}$ are positive definite design matrix, where $\kappa >0$ and $\chi >0$ are the design parameters.

Consider the following Lyapunov function of intelligent ship automatic berthing control system consisting of (1)–(2), (12) and (21)-–(23).

24 $$V=\frac{1}{2}{z}^{T}z+\frac{1}{2}{e}_{2}^{T}M{e}_{2}+\frac{1}{2}{\widetilde{W}}_{c}^{T}{\Lambda }_{c}^{-1}{\widetilde{W}}_{c}+\frac{1}{2}{\widetilde{\theta }}^{T}{\Gamma }^{-1}\widetilde{\theta }$$

Take the time derivative with respect to V, according to Eqs. (14)–(16), (19) and (21)–(23)

25 $$\begin{aligned}\dot{V}&={z}^{T}\dot{z}+{e}_{2}^{T}M{\dot{e}}_{2}-{\widetilde{W}}_{c}^{T}{\Lambda }_{c}^{-1}\dot{\widetilde{W}}-{\widetilde{\theta }}^{T}{\Gamma }^{-1}\dot{\widetilde{\theta }}=-{z}^{T}{\beta }_{1}\dot{z}-{e}_{2}^{T}M{\widehat{e}}_{2}-{e}_{2}^{T}\left({\widehat{W}}_{c}^{T}\xi \left(Z\right)+Tanh\left(\frac{{\widehat{e}}_{2}}{\sigma }\right)\widehat{\theta }\right)\\ &\quad +{e}_{2}^{T}\left({W}_{c}^{T}\xi \left(Z\right)+{d}_{c}\right)-{\widehat{e}}_{2}^{T}{W}_{c}^{T}\xi \left(Z\right)+\kappa {\widehat{W}}_{c}^{T}{\widehat{W}}_{c}-{\widetilde{\theta }}^{T}Tanh\left(\frac{{\widehat{e}}_{2}}{\sigma }\right){\widehat{e}}_{2}+\chi {\widetilde{\theta }}^{T}\widehat{\theta }\end{aligned}$$

From ${\widehat{e}}_{2}=\widehat{\nu }-\alpha$ and $\widehat{\nu }=\nu -\widetilde{\nu }$, we get ${\widehat{e}}_{2}={e}_{2}-\widetilde{\nu }$.Using lemma 2 further, we can get

26 $$-{e}_{2}^{T}{\beta }_{2}{\widehat{e}}_{2}\le -{e}_{2}^{T}\left({\beta }_{2}-\frac{1}{4}{I}_{3}\right){e}_{2}+{\Vert {\beta }_{2}\widetilde{\nu }\Vert }^{2}$$

According to formula (20)

27 $${e}_{2}^{T}{d}_{c}\le {\theta }^{T}\langle {e}_{2}\rangle \le {\theta }^{T}Tanh\left(\frac{{e}_{2}}{\sigma }\right){e}_{2}+0.2785{\sigma }^{T}\theta $$

According to formula (25) and Lemma 2, we can get

28 $$\begin{aligned}&-{e}_{2}^{T}Tanh\left(\frac{{\widehat{e}}_{2}}{\sigma }\right)\widehat{\theta }-{\widetilde{\theta }}^{T}Tanh\left(\frac{{\widehat{e}}_{2}}{\sigma }\right){\widehat{e}}_{2}+{\theta }^{T}Tanh\left(\frac{{e}_{2}}{\sigma }\right){e}_{2}={e}_{2}^{T}\left[Tanh\left(\frac{{e}_{2}}{\sigma }\right)-Tanh\left(\frac{{\widehat{e}}_{2}}{\sigma }\right)\right]\theta +{\widetilde{\theta }}^{T}Tanh\left(\frac{{\widehat{e}}_{2}}{\sigma }\right)\widetilde{\nu }\\ &\le 2{\sum }_{i=1}^{3}\left|{e}_{2,i}{\theta }_{i}\right|+{\sum }_{i=1}^{3}\left|{\widetilde{\nu }}_{i}{\widetilde{\theta }}_{i}\right|\le {e}_{2}^{T}{e}_{2}+{\Vert \theta \Vert }^{2}+\frac{{a}_{1}}{4}{\widetilde{\theta }}^{T}\widetilde{\theta }+{a}_{1}^{-1}{\Vert \widetilde{\nu }\Vert }^{2}\end{aligned}$$

where ${a}_{1}>0$ is a constant.

According to the properties of the Tanh function above, we get $Tanh\left(\frac{{e}_{2,i}}{{\sigma }_{i}}\right)\le 1$ and $Tanh\left(\frac{{e}_{2,i}}{{\sigma }_{i}}\right)-Tanh\left(\frac{{\widehat{e}}_{2,i}}{{\sigma }_{i}}\right)\le 2,i=1,2,3$. Similarly, according to formula (28) we get.

$${e}_{2}^{T}Tanh\left(\frac{{e}_{2}}{\sigma }\right)-Tanh\left(\frac{{\widehat{e}}_{2}}{\sigma }\right)\le 2{\sum }_{i=1}^{3}\left|{e}_{2,i}{\theta }_{i}\right|, \ {\widetilde{\theta }}^{T}Tanh\left(\frac{{\widehat{e}}_{2}}{\sigma }\right)\widetilde{\nu }\le {\sum }_{i=1}^{3}\left|{\widetilde{\nu }}_{i}{\widetilde{\theta }}_{i}\right|.$$

According to the Lemma 2–3, ${\widehat{e}}_{2}={e}_{2}-\widetilde{\nu }$ and ${\widetilde{W}}_{c}={W}_{c}-{\widehat{W}}_{c}$, we get

29 $$\begin{aligned}&-{\widehat{e}}_{2}^{T}{\widehat{W}}_{c}^{T}\xi \left(\widehat{Z}\right)+{e}_{2}^{T}{W}_{c}^{T}\xi \left(Z\right)-{\widehat{e}}_{2}^{T}{\widetilde{W}}_{c}^{T}\xi \left(\widehat{Z}\right)={e}_{2}^{T}{W}_{c}^{T}\left[\xi \left(Z\right)-\xi \left(\widehat{Z}\right)\right]+{\widetilde{\nu }}^{T}{\widetilde{W}}_{c}^{T}\xi \left(\widehat{Z}\right) \\ &\le \frac{{e}_{2}^{T}{e}_{2}}{2}+\frac{{W}_{m}^{2}{\delta }^{2}{\kappa }^{2}}{2}+\frac{{b}_{1}}{4}{\widetilde{W}}_{c}^{T}{\widetilde{W}}_{c}+\frac{1}{{b}_{1}}{\Vert \widetilde{\nu }\Vert }^{2}{\Vert \xi \left(\widehat{Z}\right)\Vert }^{2}\end{aligned}$$

where ${b}_{1}>0$ is a constant, and ${b}_{1}$ is used only for stability analysis.

According to Lemma 2, the following inequality is true

30 $${\widetilde{W}}_{c}^{T}{\widetilde{W}}_{c}\le -\frac{3}{4}{\widetilde{W}}_{c}^{T}{\widetilde{W}}_{c}+{W}_{m}^{2}$$

31 $${\widetilde{\theta }}^{T}\widehat{\theta }\le -\frac{3}{4}{\widetilde{\theta }}^{T}\widehat{\theta }+{\Vert \theta \Vert }^{2}$$

Substitute Eq. (26)–(31) into Eq. (25) to get

$$ \begin{aligned} \dot{V} & \le - z^{T} \beta_{1} \dot{z} - e_{2}^{T} \left( {\beta_{2} - \frac{7}{4}I_{3} } \right)e_{2} - \frac{{3\kappa - b_{1} }}{4}\tilde{W}_{c}^{T} \tilde{W}_{c} { } \\ & \quad - \frac{{3\chi - a_{1} }}{4}\tilde{\theta }^{T} \tilde{\theta } + 2||\theta ||^{2} + \frac{1}{2}W_{m}^{2} \delta^{2} \overline{\kappa }^{2} + ||\beta_{2} \tilde{\nu }||^{2} { } \\ \end{aligned} $$

32 $$+{W}_{m}^{2}+{a}_{1}^{-1}{\Vert \widetilde{\nu }\Vert }^{2}+{b}_{1}^{-1}{\Vert \widetilde{\nu }\Vert }^{2}{\Vert \xi \left(\widehat{Z}\right)\Vert }^{2}+0.2785{\sigma }^{T}\theta $$

According to Theorem 1, $\widetilde{\nu }$ is bounded. Furthermore, the properties of lemma 2 and the Gaussian function show that $\Vert \xi \left(\widehat{Z}\right)\Vert$ is bounded. There is also a normal number $\Theta$ making ${\Vert {\beta }_{2}\widetilde{\nu }\Vert }^{2}+{a}_{1}^{-1}{\Vert \widetilde{\nu }\Vert }^{2}+{b}_{1}^{-1}{\Vert \widetilde{\nu }\Vert }^{2}{\Vert \xi \left(\widehat{Z}\right)\Vert }^{2}\le \Theta$. Therefore, Eq. (32) can be rewritten as

33 $$\begin{aligned}\dot{V}&\le -{z}^{T}{\beta }_{1}\dot{z}-{e}_{2}^{T}\left({\beta }_{2}-\frac{7}{4}{I}_{3}\right){e}_{2}-\frac{3\kappa -{b}_{1}}{4}{\widetilde{W}}_{c}^{T}{\widetilde{W}}_{c}-\frac{3\chi -{a}_{1}}{4}{\widetilde{\theta }}^{T}\widetilde{\theta }+2{\Vert \theta \Vert }^{2}+\frac{1}{2}{W}_{m}^{2}{\delta }^{2}{\bar{\kappa }}^{2}+\Theta +{W}_{m}^{2}+0.2785{\sigma }^{T}\theta \\ &\le -{\lambda }_{min}\left({\beta }_{1}\right){z}^{T}z-\left({\lambda }_{min}\left({\beta }_{2}\right)-\frac{7}{4}\right){\widetilde{m}}^{-1}{e}_{2}^{T}M{e}_{2}-\frac{3\kappa -{b}_{1}}{4}{\lambda }_{min}\left({\Lambda }_{c}\right){\widetilde{W}}_{c}^{T}{\Lambda }_{c}^{-1}{\widetilde{W}}_{C}-\frac{3\chi -{\alpha }_{1}}{4}{\lambda }_{min}\left(\Gamma \right){\widetilde{\theta }}^{T}{\Gamma }^{-1}\widetilde{\uptheta }\\ &\quad +2{\Vert \theta \Vert }^{2}+\frac{1}{2}{W}_{m}^{2}{\delta }^{2}{\overline{\kappa }}^{2}+\Theta +{W}_{m}^{2}+0.2785{\sigma }^{T}\theta \le -\varpi V+\mu\end{aligned} $$

where

33 $$\varpi =min\left\{2{\lambda }_{min}\left({\beta }_{1}\right), 2\left({\lambda }_{min}\left({\beta }_{2}\right)-\frac{7}{4}\right),\frac{3\kappa -{b}_{1}}{4}{\lambda }_{min}\left({\Lambda }_{c}\right),\frac{3\chi -{\alpha }_{1}}{4}{\lambda }_{min}\left(\Gamma \right)\right\}$$

34 $$\mu =2{\Vert \theta \Vert }^{2}+\frac{1}{2}{W}_{m}^{2}{\delta }^{2}{\bar{\kappa }}^{2}+\Theta +{W}_{m}^{2}+0.2785{\sigma }^{T}\theta $$

In addition, design parameters ${\beta }_{2},\kappa,\chi$ are satisfied

35 $${\lambda }_{min}\left({\beta }_{2}\right)>\frac{7}{4}$$

36 $$\kappa >\frac{{b}_{1}}{3}$$

37 $$\chi >\frac{{a}_{1}}{3}$$

### Theorem 2

Considering that the smart ship described in formula (1)–(2) is affected by dynamic uncertainty and external interference under hypothesis 1–3 and initial conditions $\left|{e}_{1,i}\left(0\right)\right|<{\sigma }_{i}\left(0\right)$, the design control law (21) and adaptive law (22)–(23), the virtual control vector (15), the design control law (21), the design control law (15), and the design control law (15).The nonlinear transformation (12) enables the surface intelligent vessel to complete the berthing operation according to the predetermined error range, while ensuring that all signals in the automatic berthing control system are uniformly and ultimately bounded, and that the berthing control error $\eta -{\eta }_{d}$ converges to the predefined residual set in a predefined time.

According to Theorem 2, all signals of automatic berthing control closed loop system (1)–(2) in control law (21) and adaptive law (22)–(23) are uniformly bounded.

Prove as follows:

Equation (33) can be written as

38 $$V\le \frac{\mu }{\varpi }+\left(V\left(0\right)-\frac{\mu }{\varpi }\right){e}^{-\varpi \mu }$$

where $V\left(0\right)$ is the initial value of $V$.

We know from Eq. (33) that $V$ is bounded. So, since Eq. (24), ${e}_{2},z,{\widetilde{W}}_{c}$ is also bounded. Also, since ${\widetilde{W}}_{c}={W}_{c}-{\widehat{W}}_{c}$ and $\widetilde{\theta }=\theta -\widehat{\theta }$, ${\widetilde{W}}_{c}$ and $\widetilde{\theta }$ are also bounded. Further, we get the boundedness of $\alpha$ in formula (15) based on assumptions 3 and ${e}_{1}=\eta -{\eta }_{d}$ , and the boundedness of $\alpha$ based on the boundedness of formula (16). Given the boundedness of $\widetilde{v}$ and ${\widehat{e}}_{2}={e}_{2}-\widetilde{v}$, ${\widehat{e}}_{2}$ is bounded. Therefore, $\tau$ in formula (20) is bounded. Based on the above analysis, all signals in the automatic berthing closed-loop control system are uniformly and ultimately bounded. Further, since ${\mathit{lim}}_{t\to \infty }{e}^{-\varpi t}=0$ .$Z$ converges to compact set $\Omega =\left\{z\in {R}^{3},\Vert z\Vert \le \sqrt{2\mu /\varpi }\right\}$, by selecting the design parameters of ${\beta }_{1},{\beta }_{2},{\Lambda }_{c},\Gamma ,\kappa ,\chi$ to arbitrarily small to ensure formula (36)–(38) is founded. In addition, according to formula (12), it can be found that $\left|{e}_{1,i}\right|<{\sigma }_{i}$ is valid for $\forall t>0$, that is, formula (11) is valid. Thus, due to the properties of the predetermined performance time function, the berthing error ${e}_{1}$ can converge to a predefined residual set (28) in a predefined time. Theorem 2 is proved.

## Simulation

In order to verify the effectiveness and superiority of the berthing control scheme with predetermined performance proposed in this paper under the influence of uncertain dynamics and external disturbances, it is compared with the control scheme designed in reference^22^. The comparative control scheme (FTPF) designs finite time parameters in the control law and virtual control law to achieve finite time control, while the control scheme (PTPF) used in this paper achieves predetermined performance control by designing predetermined performance time parameters (Table 2). CyberShip2, a 1:70 scale supply ship model from the Norwegian University of Science and Technology, was selected as the simulation object, with a mass of 23.8 kg, a length of 1.255 m and a width of 0.29 m.The relevant hydrodynamic parameters are described in reference^17^.

**Table 2:** Design Parameters.

Project	Value
$\kappa$	0.04
$\chi$	0.08
${\sigma }_{u}$	0.01
${\sigma }_{v}$	0.01
${\sigma }_{r}$	0.02
${\Lambda }_{c}$	[30 0 0;0 30 0;0 0 30]
$\Gamma$	[1 0 0;0 1.5 0;0 0 1.5]
${\beta }_{1}$	[0.5 0 0;0 0.5 0;0 0 0.5]
${\beta }_{2}$	[40 0 0;0 40 0;0 0 40]
${l}_{i},i=1,2,3$	1; 0.5; 1
${h}_{i},i=1,2,3$	6; 18; 0.5
${T}_{f,i},i=1,2,3$	60; 20; 30
$\sigma {T}_{f,i},i=1,2,3$	0.1; 0.1; 20

In the simulation experiment, the initial state parameters of ship berthing are selected as:

$x\left(0\right)=-10/L$, $y\left(0\right)=-10/L$, $\psi \left(0\right)=\frac{\pi }{3}{\text{rad}}$, $u\left(0\right)=0.5\text{m/}s$, $v\left(0\right)=0\text{m/}s$, $r\left(0\right)=0\text{rad/s}$, The target berthing states are ${x}_{d}=0/L$, ${y}_{d}=0/L$, ${\psi }_{d}=0rad$.External interference ${\tau }_{du}$,${\tau }_{dv}$, and ${\tau }_{dr}$ are set to

39 $$\left\{\begin{array}{c}{\tau }_{du}=1.1\left({\text{sin}}\left(0.02\pi t+\frac{\pi }{4}\right)+{\text{cos}}\left(0.01\pi t\right)\right)\\ {\tau }_{dv}=0.4\left({\text{cos}}\left(0.02\pi t-\frac{\pi }{8}\right)-{\text{sin}}\left(0.05\pi t\right)\right)\\ {\tau }_{dr}=0.8\left({\text{sin}}\left(0.01\pi t+\frac{\pi }{3}\right)+{\text{cos}}\left(0.01\pi t\right)\right)\end{array}\right.$$

**Figure 2:**
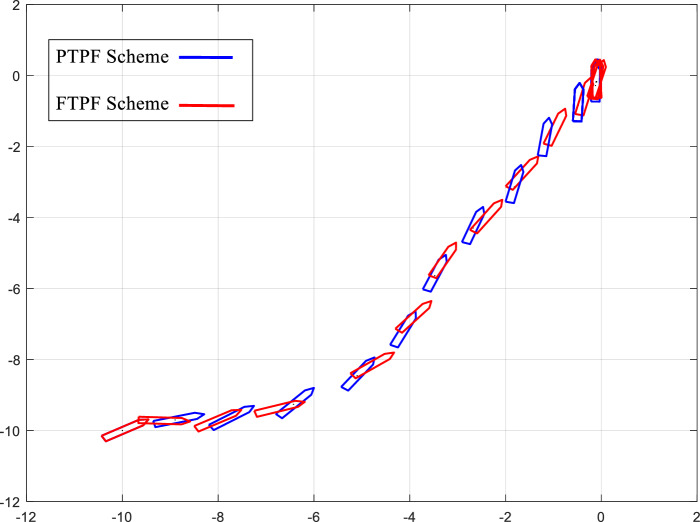
Design and comparison plan.

**Figure 3:**
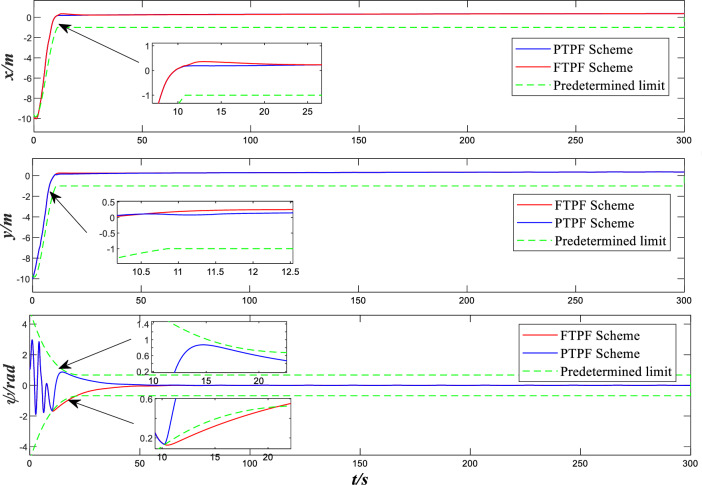
Design and Comparison positions and yaw angles.

Figure 2 shows the trajectory of automatic berthing. It can be seen from Fig. 2 that in the whole control process, although the trajectory near the comparison scheme is similar to the predetermined performance control scheme, the heading angle of the comparison scheme changes irregularly and has a large amplitude, which cannot meet the predetermined limit designed by us (as shown in the dashed line in Fig. 3).

Figure 3 shows the positions and yaw angles error curve of with time. It can be seen from the figure that both control methods can complete the control task. Both the comparison control method using adaptive neural network only and the control method introducing predefined performance time function in this paper reach stability around 15 s. However, within the interval approaching stability, there is a gap of about 0.1 m between the two schemes in the x and y directions, and the gap is even larger for φ. Compared with the control scheme proposed in this paper, the comparison control scheme has exceeded the predetermined limit before approaching the stability point. Since pre-defined performance time function is adopted to optimize transient performance, the control scheme adopted in this paper has better control effect than the comparison control scheme before stability is reached. As can be seen from the figure, the approach trend of is more stable before stability is reached, with no obvious overshoot.

**Figure 4:**
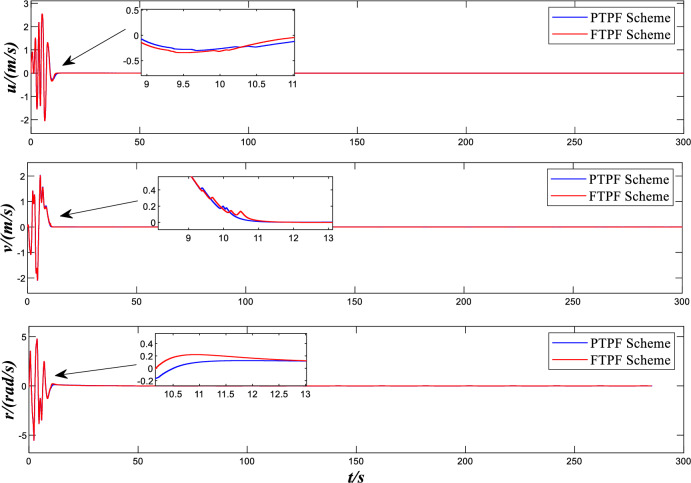
Design and Comparison speed and yaw rates.

Figure 4 shows the speed and yaw rates curve of over time. It can be seen from the graph that no matter the predefined time control scheme or the comparison control scheme designed in this paper, the ship velocity vector tends to be stable at around 20 s and has a reasonable error range. However, before stability is achieved, it can be seen from the figure that the comparative control scheme has a larger mutation amplitude before stability, and the control effect is relatively worse. However, compared with the control scheme designed in this paper, the number of mutations before reaching stability is 1 less. By comparing the two control schemes, it is not difficult to see that after reaching a relatively stable state, the comparison control scheme has chattering phenomenon for a period of time, while the control scheme designed in this paper maintains better steady-state performance.

**Figure 5:**
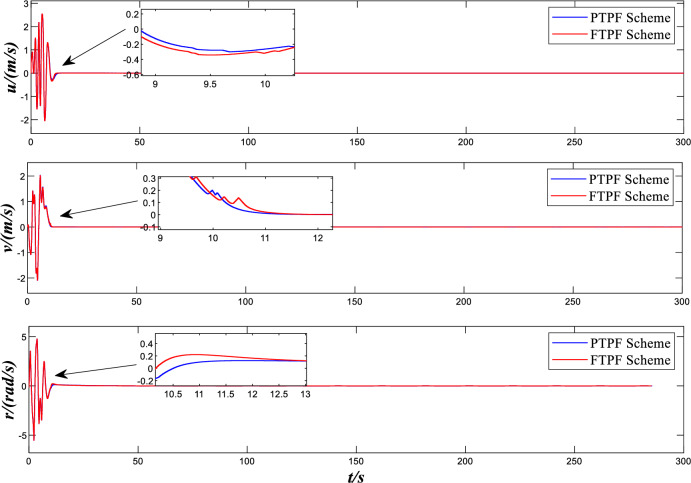
Control inputs.

Figure 5 shows the time response curves of forward force ${\tau }_{u}$, transverse drift force ${\tau }_{v}$ and yawing moment ${\tau }_{r}$ under the PTPF scheme designed in this paper and the contrast control scheme. Considering the actual situation of the simulated ship model, the maximum output power of the ship is set to $\pm 20N$ .It can be clearly seen that the actual control input of both the PTPF scheme and the contrast control scheme can ensure a relatively stable state. In the stable process, the control scheme designed in this paper not only has a small number of control force mutations and a small control peak when the mutation occurs, but also the comparison control scheme will still have chattering after reaching a relatively stable state. However, this further indicates that the PTPF control scheme in this paper has excellent steady-state performance and strong robustness.

## Conclusion

In this paper, a pre-defined performance time function is designed to solve the problem of ship automatic berthing control which is affected by both external interference and model uncertainty, and the tracking error and the pre-defined performance time function are coupled into the transformation error. Secondly, the neural network technology is used to approximate the model uncertainty online, and the model uncertainty and external interference are simulated to synthesize a virtual composite parameter, and the adaptive technology is combined with the virtual parameter learning method to estimate and compensate the virtual composite parameter online. Finally, under the environment of external interference and dynamic influence of model uncertainty, simulation analysis and comparison experiment are carried out to verify the effectiveness of the controller proposed in this paper. By analyzing the simulation results, it can be concluded that the control scheme designed in this paper has superior control performance and steady-state performance under the dynamic influence of external interference and model uncertainty at the same time, and can control the error in a pre-defined residual set to achieve better transient performance. It is worth noting that in order to ensure the control effect, the control scheme designed in this paper has a drastic change in heading angle, and the optimization algorithm will strive to solve this problem in the future.

## Data Availability

The datasets used and/or analysed during the current study available from the corresponding author on reasonable request.
